# The Peritoneum as a Natural Scaffold for Vascular Regeneration

**DOI:** 10.1371/journal.pone.0033557

**Published:** 2012-03-16

**Authors:** Stefano Bonvini, Mattia Albiero, Luca Ferretto, Annalisa Angelini, Piero Battocchio, Marny Fedrigo, Michele Piazza, Gaetano Thiene, Angelo Avogaro, Gian Paolo Fadini, Franco Grego

**Affiliations:** 1 Vascular and Endovascular Surgery, Department of Cardiac, Thoracic and Vascular Sciences., University of Padova, Padova, Italy; 2 Department of Medicine, University of Padova, Padova, Italy; 3 Laboratory of Experimental Diabetology, Venetian Institute of Molecular Medicine, Padova, Italy; 4 Cardiovascular Pathology, Department of Cardiac, Thoracic and Vascular Sciences, University of Padova, Padova, Italy; Bristol Heart Institute, University of Bristol, United Kingdom

## Abstract

**Objective:**

The peritoneum has the same developmental origin as blood vessels, is highly reactive and poorly thrombogenic. We hypothesize that parietal peritoneum can sustain development and regeneration of new vessels.

**Methods and Results:**

The study comprised two experimental approaches. First, to test surgical feasibility and efficacy of the peritoneal vascular autograft, we set up an autologous transplantation procedure in pigs, where a tubularized parietal peritoneal graft was covered with a metal mesh and anastomosed end-to-end in the infrarenal aorta. Second, to dissect the contribution of graft vs host cells to the newly developed vessel wall, we performed human-to-rat peritoneal patch grafting in the abdominal aorta and examined the origin of endothelial and smooth muscle cells. In pig experiments, the graft remodeled to an apparently normal blood vessel, without thrombosis. Histology confirmed arterialization of the graft with complete endothelial coverage and neointimal hyperplasia in the absence of erosion, inflammation or thrombosis. In rats, immunostaining for human mitochondri revealed that endothelial cells and smooth muscle cells rarely were of human origin. Remodeling of the graft was mainly attributable to local cells with no clear evidence of c-kit+ endothelial progenitor cells or c-kit+ resident perivascular progenitor cells.

**Conclusions:**

The parietal peritoneum can be feasibly used as a scaffold to sustain the regeneration of blood vessels, which appears to occur through the contribution of host-derived resident mature cells.

## Introduction

Atherosclerotic vascular disease often leads to critical narrowing or extensive aneurysmatic dilation of the vessel lumen. In these circumstances, surgical techniques use biocompatible materials to by-pass the stenosis or repair the aneurysm. Currently, this is accomplished using arterial or venous autografts, when suitable. For the reconstruction of larger vessels, such as the aorta, autografts with adequate caliber and resistance are not available. In this case, allografts or synthetic grafts are commonly used. However, there are pitfalls in these approaches as homografts tend to degenerate over time, while synthetic grafts are thrombogenic and prone to infection [1], [2], [3].

On this basis, several laboratories have development tissue-engineered blood vessels *in vivo* or *in vitro* using molds of prosthetic or biodegradable scaffolds, but each artificial graft has shown significant limitations [4]. Moreover, the production of a bioengineered graft is time-consuming, and unsuitable in emergency or where qualified laboratories are lacking.

We hypothesize that the peritoneum represents an ideal inner layer for a vascular substitute, as it is a highly reactive and readily available tissue, with the same embryologic origin as the vasculature (the mesoderm). The peritoneum is composed of a mesothelial layer supported by a thin connective tissue containing blood vessels, lymphatics and nerves [5]. Owing to its biological characteristics, the peritoneum has been proposed as an alternative for the coating of vascular prostheses [5], [6], [7]. In an experimental study on rabbits, Sparks et al. have demonstrated that prosthetic grafts coated with mesothelial cells have a higher patency rate if compared to uncoated grafts [8]. According to Campbell et al. the peritoneum seems to offer a lower risk of thrombosis than synthetic grafts [9]. The anticoagulant properties of the peritoneum have been demonstrated in animal models using a peritoneum/fascia patch on pulmonary arteries as substitute of pericardium [10]. For these reasons, the parietal peritoneum has been proposed as a vascular substitute. For instance, Sarac et al. studied a fascia-peritoneum patch as a pledget for an infected aortic stump [11] and as an arterial substitute in femoral artery patch angioplasty on dogs [12]. Garcia-Graz et al. [13] created a vascular autograft with the posterior rectus aponeurosis including the subjacent peritoneum in seven dogs, and performed an implantation on the aorta by an end-to-end surgical anastomosis; to avoid the dilatation of the graft the authors encircled the fasciaperitoneum with three rings made of a 3/0 silk floss. Their results seem to demonstrate the feasibility of this approach, with impermeability and no thrombogenicity, but the study lacked of histological examination.

Structural weakness and low resistance to stress and pulsatile pressure are the major limitations in using the peritoneum as an arterial graft, which can be circumvented using a mechanical support to avoid deformation of the graft. As several investigations suggest that hemodynamic forces are a stimulus for tissue remodeling and acquisition of a contractile phenotype [9], [14], propagation of the sphygmic wave by a metal mesh may allow the peritoneum to differentiate in an artery-like structure.

The aim of the present study was to evaluate the feasibility and efficacy of a substitute vascular graft, made by a tubularized peritoneal flap covered with a metal mesh. We also aimed to understand the role of cells derived from the peritoneum in the arterialization of the vascular graft, to distinguish cell differentiation from scaffold function.

## Materials and Methods

### Ethics statement

The animal protocols used in this study were reviewed and approved by the animal care section of the Ministry of Health (n° 3262/2009-A and 87/2010-B). The Center for Experimental Surgery of the University of Padova specifically approved the use of human tissues. Written informed consent was acquired from human subjects involved in the study.

### Pig autologous tabularized peritoneal aortic graft

**Figure 1:**
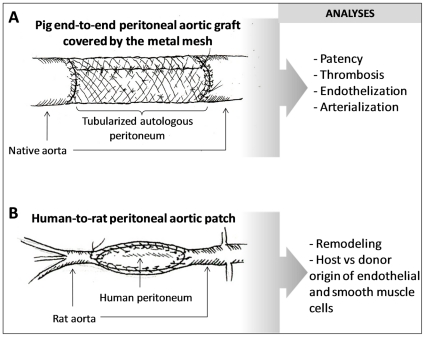
Graphic representation of the two types of experiments. A) A pig autologous end-to-end peritoneal implant was used to study patency, eventual thrombosis, endothelization and arterializations of the graft, 2 weeks after surgery. B) A human-to-rat peritoneal graft approach was set up to study remodeling of the peritoneum and to determine the host vs donor origin of cells that contributed to the process of endothelization and eventual arterialization.

Two types of experiments were set up to study the patency, eventual thrombosis, endothelization and arterialization of the graft, as well as the origin of cells that contributed to its remodeling (Figure 1). Five 30 days old female pigs underwent induction with sodium thiopental and general anesthesia (1 mg/kg Xilazine plus 0.5 mg/kg Tiletamine chlorhydrate) with endotracheal intubation. The abdominal cavity was accessed with a midline incision. A 5×3 cm rectangular layer of the abdominal peritoneum was collected, leaving in situ the posterior rectus aponeurosis. The peritoneum was tubularized on a plastic tutor by a continuous 6/0 polypropylene suture, creating a 10 mm diameter, 5 cm length vascular graft. The intra-abdominal surface of the peritoneum was used as the internal sheath of the graft. Then, the peritoneum tubularized on the tutor was covered with a stainless steel mesh (Biocompound Shunt® - Alpha Research Switzerland). After dissection of the infrarenal aorta, intravenous sodium heparin (150 IU/kg) was administrated, and the aorta was clamped. A 4 cm portion of the infrarenal aorta was removed and the mesh/peritoneal autograft was inserted by end-to-end surgical anastomosis, performed with 6/0 polypropylene thread. The removed aortic portion must be 1 cm shorter than the vascular autograft because the aorta is very elastic and retracts. Patency and resistance of the graft was confirmed by the visual intra-operative inspection and pulse-checking of the proximal and distal aorta. Then, optimal hemostasis was verified and the abdomen was closed by layers. During the post-operative period, each animal received analgesic medication (fentanyl 0.1 mg/day), antibiotics and a daily subcutaneous injection of 6000 IU of low-molecular-weight-heparin (enoxaparin). Animals were observed for 2 weeks, evaluating clinical parameters and eventual development of arterial insufficiency (intensity of pulse, temperature, trophism and functional changes of the hind limbs). One week after surgery, an echo-color-Doppler scan was performed under pharmacological sedation to confirm patency of the graft. Two weeks after surgery, animals were sacrificed under general anesthesia. A re-do open transperitoneal approach to the aorta was performed and autografts, including the whole proximal and distal anastomosis, were harvested.

### Human-to-rat peritoneal aortic patch

Ten week old male Sprague-Dawley rats (n = 3), weighing 200 to 250 g, from the University animal house vivarium were used for the experiment. The experimental protocols were approved by the Animal Ethics Committee of the University of Padova. Rats were housed in individual cages in a temperature and light-dark cycle-controlled environment with free access to food and water. All rats received standard care, in compliance with the “Principles of Laboratory Animal Care” prepared by the Institute Experimental Surgery of the University of Padova. The rats were anesthetized with intramuscular injection of 700 µg/kg tilethamine hydrochloride – zolazepam hydrochloride mixture plus 150 µg/kg xylazine subcutaneously, and then placed on a supine position under a heating lamp. The skin was aseptically prepared and a midline laparotomy was done. Ten mL of warm normal saline was instilled into the peritoneum cavity to help maintain fluid balance. The abdominal aorta was exposed by gently deflecting the loops of intestine to the left with moist gauze swabs and the infra renal aorta was isolated till the iliac origin. Atraumatic microvacsular clamps (Vascu-Statts II, midi straight 1001-532; Scanlan Int., St. Paul, MN) were placed on the aorta just below the origin of renal arteries and on the aortic carrefour. A longitudinal aortotomy of 1 cm was performed between the clamps. The aortotomy was then closed using a patch of human peritoneum harvested the same day and sutured with separated stitches of polypropylene 8/0. The patency of the aorta distally to the patch was tested by visual intra-operative inspection and pulse-checking. The hemostasis was verified and the abdomen closed. The donors of the peritoneum gave written informed consent but the sample was collected in an anonymous way. In rats, a tabularized end-to-end peritoneal graft could not be performed because a suitable caliber metal mesh was not available.

### Histology and immunohistochemistry

#### Pig samples

Multiple cross-sections of the aorta and the graft were embedded in paraffin after formalin fixation. Double immunohistochemical staining was performed for alpha smooth muscle actin (mouse monoclonal antibody, clone 1A-4, dilution 1∶50, Dako) and elastic van Gieson fibres to identify tissue composition and vessel wall remodeling as well as for von Willebrand factor (rabbit polyclonal antibody, dilution 1∶100, Biocare Medical) to stain the endothelium.

#### Rat samples

A 2 cm long sample of abdominal aorta was infused and embedded with OCT (BioOptica, Milan, Italy) and then frozen in liquid nitrogen. All stainings were performed on 10 µm-thick cryosections. Hematoxylin-eosin staining was performed according to the manufacture's instruction (BioOptica). Immunofluorescence stainings were performed with mouse anti-smooth muscle alpha actin (dilution 1∶100, Sigma Aldrich, St. Louis, MO, USA), or rabbit anti-human von Willebrand factor (dilution 1∶100, Dako, Sweden), anti-c-kit (dilution 1∶100, BD Biosciences), anti-CD140b (dilution 1∶100, BD Biosciences), anti-NG2 (dilution 1∶100, R&D Systems), or mouse anti-human mitochondria (dilution 1∶50, Abcam, Cambridge, UK). As secondary antibodies we used Cy2-conjugated goat anti-rabbit antibody (dilution 1∶150, Millipore, Billerica, MA), or Alexa Fluor®594-conjugated goat anti-mouse antibody (dilution 1∶150, Invitrogen, Carlsbad, CA, USA), or DyLight488-cnojugated goat anti-mouse antibody (dilution 1∶150, Jackson ImmunoResearch Laboratories, Inc., West Grove, PA, USA). Antibodies were incubated in 1% PBS/BSA solution for 30 minutes at 37°C. Nuclei were counterstained with Hoechst 33258 (Sigma Aldrich).

## Results and Discussion

### Arterialization of a tabularized peritoneal aortic autograft in pigs

**Figure 2:**
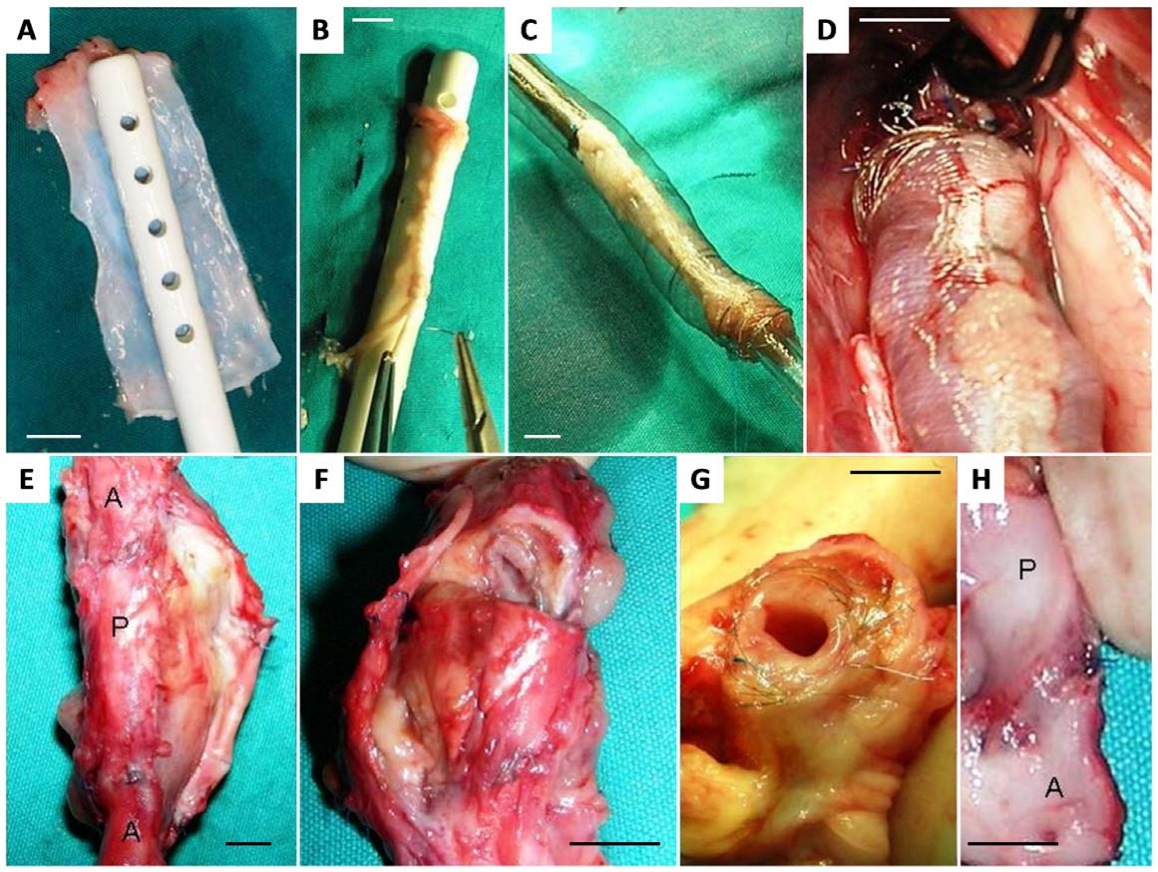
Macroscopic representation of graft preparation, implantation and removal in pigs. A–B) Tailoring of the peritoneal graft on a tutor. C) Coverage of the peritoneal graft with the metal mesh. D) Proximal anastomosis showing the adhesion of the peritoneum to the metal mesh when pulsatile flow is established into the graft. E) Graft harvested (A, native aorta; P, peritoenal graft) with no signs of overt dilatation. F) Fresh transversal equatorial cut of the peritoneal graft showing patency and (G) thickening of the peritoneal graft at 15 days from the implant. H) Intraluminal appearance at the proximal anastomosis (A, native aorta; P, peritoenum graft). Scale bars 1 cm.

**Figure 3:**
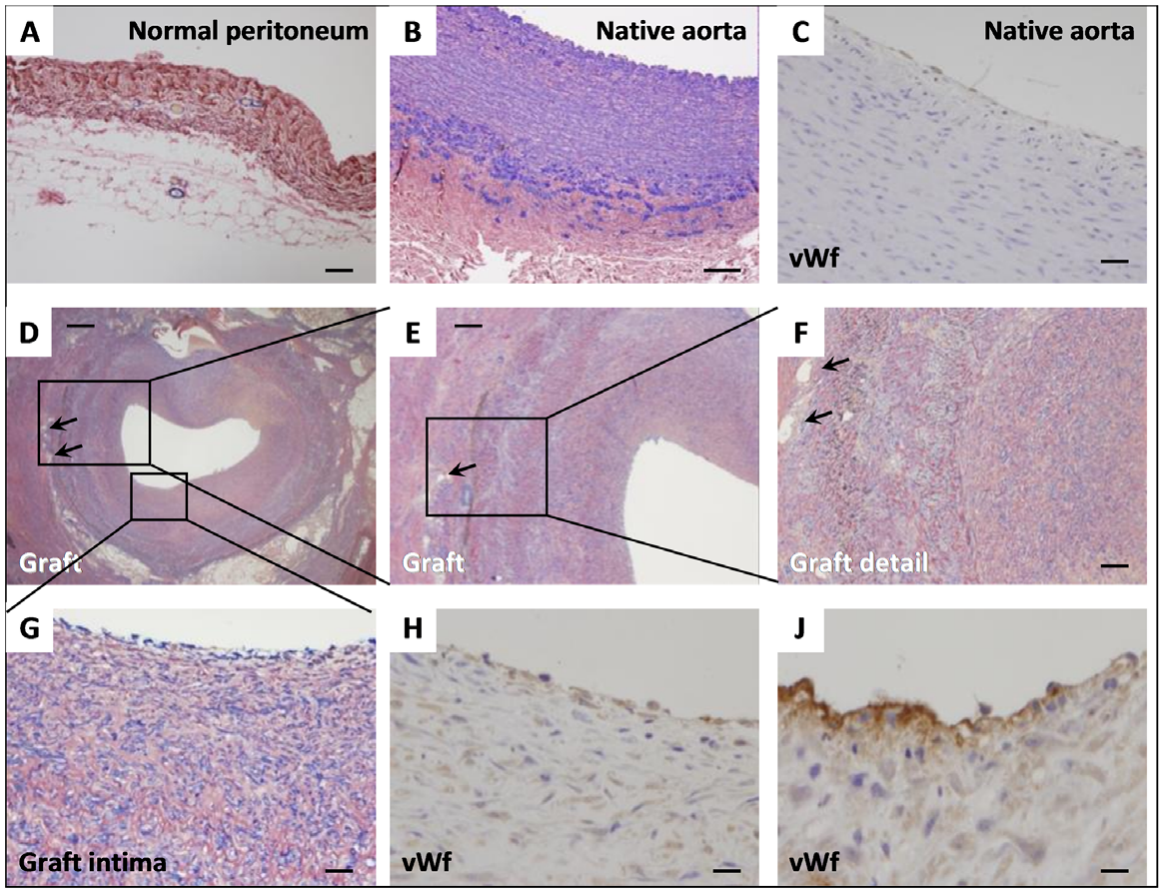
Histopathological analysis of the peritoneal patch explanted from pigs. Peritoneal graft pre-innestum (A), native aorta (B, C) and graft after two weeks (D–J). A) normal peritoneum, double staining with alfa-SMA (blue) and elastic von Gieson (black-brown) magnification 80×, scale bar 40 µm. B) native aorta, double staining showing the media with elastic fibres in black and smooth muscle cells in blue, magnification 50×, scale bar 50 µm. C) Native aorta, von Willebrand factor staining of the endothelium, magnification 160×, scale bar 20 µm. D) Graft and metallic scaffold (arrows indicate some of the holes around the graft), magnification 12,5×, scale bar 250 µm. E) graft remodeling, magnification 31× scale bar 100 µm. F) Arterialization of graft, magnification 62,5×, scale bar 50 µm. G) Intimal hyperplasia, magnification 125×, scale bar 25 µm. H, I) Endothelization of the graft, von Willebrand factor staining magnification 160× (scale bar 20 µm) and 320× (scale bar 10 µm), respectively.

All implantation procedures were uneventful and pigs survived until the pre-specified time point for sacrifice (2 weeks). No technical complication occurred during preparation of the peritoneal graft (Figure 2A–D), which took about 25 minutes. The mean operative time was about 150 minutes, without significant blood loss. All the animals remained hemodynamically stable during surgery. Impermeability was satisfactory and there was no bleeding or embolization. In two cases, a single 6/0 polypropilene stitch on proximal anastomosis was necessary to obtain a perfect hemostasis. All pigs had a normal postoperative recovery, with an increase in body weight of about 8.5 kg. During the abdominal re-intervention no periaortic hematomas or false aneurysms were noticed. Patency and absence of stenosis were checked by an intraoperative echo-color-Doppler scan. After removal of the grafts, it was possible to confirm that the metal mesh was still in situ, maintaining a good shape and providing an optimal resistance to passive dilatation (Figure 2E). Macroscopically, metallic scaffolds were inside the peri-adventitial fibrous tissue and the peritoneum inside the graft appeared vital, with no sign of surface lesion or thrombosis (Figure 2F–G). No discontinuity were visible in the intima of native aortas as well as of the implanted graft (Figure 2H). The peritoneal graft appeared to have thickened compared with its original shape, reaching roughly the same thickness as the aortic wall (aorta 1.8±0.5 mm; patch 1.7±0.2 mm). Histological evaluation confirmed the arterialization process within the peritoneal tube with neointimal hyperplasia, periadventitial reactive fibrosis and neoangiogenesis. The peritoneal tube, normally composed by collagen, a few blood vessels and adipose tissue (Figure 3A), appeared to have functioned as a scaffold and was colonized by myofibroblasts (Figure 3D–G). Intimal hyperplasia was characterized by smooth muscle cell proliferation (alfa-SMA positive staining) with a typical feature of fusated or stellated cells in the absence of inflammation or thrombosis (Figure 2E–G). The graft wall was thickened (mean 0.66±0.2 mm) because of intimal hyperplasia, proliferation of myofibroblast and neovascularization of adventitia around the metallic scaffold (holes in Figure 3D–F). In addition, a chronic inflammatory infiltrate, including lymphocytes, macrophages, multinucleated giant cells was suggestive of foreign body reaction between metallic scaffold and adventitia (Figure 3E–G). Immunostaining for von Willebrand factor showed the complete endothelization of the peritoneal graft (Figure 3H–J). In view of a hypothetical use of the peritoneal graft as a vascular substitute in humans, the occurrence of excess neo-intimal thickening may represent a limitation. However, the remodeling was mainly ad-luminal, as the cross-sectional lumen area of the graft was similar to that of the native aorta. The extensive cell density developed within the neo-intimal area is likely a result of the high tissue reactivity of this animal model. Indeed, pigs were in their rapid growth curve phase and gained more than 500 g a day. This is also the reason why the experiment could not be prolonged beyond the pre-specified 2 week time point. A longer period of observation would create a mismatch between the growing caliber of the native aorta and the fixed caliber of the metal mesh covering the peritoneal graft.

### Cells that contribute to arterialization of the peritoneal graft are mainly of host origin

The peritoneum develops from the coelom mesoderm and includes cells of mesenchymal origin, which have the capacity to differentiate into several cell types. Indeed, the presence of pluripotent mesenchymal cells within the peritoneum raises the possibility of using the peritoneal mesothelium in regenerative therapy. Peritoneal mesothelial cells are endowed with such a degree of plasticity that, if placed in the appropriate microenvironment they have a remarkable potential to generate other cell-lines [15], [16]. Owing to this property, a gene-modified peritoneal cell patch has been recently used to promote healing of experimental myocardial infarction [17].

To understand whether cells contributing to remodeling and arterialization derived from the graft or from local/circulating cells, we set up a human-to-rat peritoneal xenografing procedure, in which a small fragment of human parietal peritoneum was used as a patch at the site of infrarenal aortic arteriotomy in rats. We then identified human cells by staining with a specific anti-human mitochondri, which does not react with rat antigens. The surgical procedure was uneventful. At time of sacrifice, the rat abdominal aorta with the human peritoneal patch appeared patent, without signs of thrombosis. A dense tissue was found around the patched peritoneum. At transversal cut, the lumen of the patched aorta was non-significantly increased compared to that of the normal downstream aorta (mean diameter 1.6±0.3 vs 1.3±0.2 mm).

**Figure 4:**
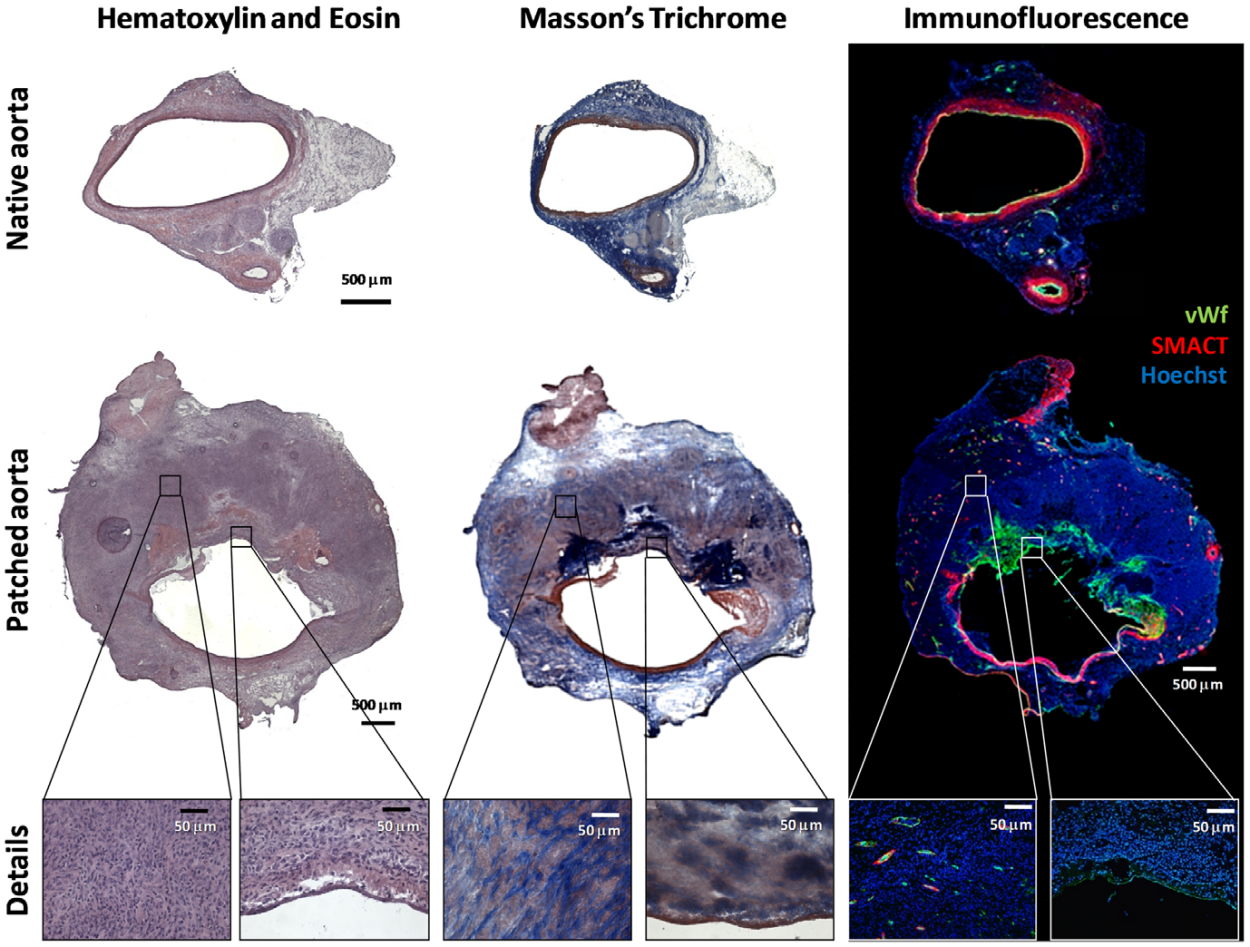
Histopathological analysis of the aortic peritoneal patch explanted from rats. Sections of the native aorta (top panels) and the patched aorta (middle panels and details in the bottom panels), were stained with hematoxylin and eosin, Masson's trichrome and using immunofluorescence for endothelium (vWf) and smooth muscle cells (alpha-SMA). The peritoneal patch (at the top) was covered by an endothelial layer, but there was no tunica media (red staining). Compared with the native aorta, the transversal sections containing the peritoneal patch showed extensive thickening, formed by a densely cellular reactive tissue. Detail panels in the bottom of the figure show that such tissue contained elongated cells, collagen, as well as capillaries and arterioles.

Histopathological analysis revealed complete endothelization of the patch (Figure 4). There was significant neo-intimal thickening, composed mainly by cells that were negative for alpha-SMA, and by vWf-expressing endothelial cells. The rare alpha-SMA-expressing cells in the patch site were dispersed and not organized as a medial layer. The surrounding neo-formed tissue had a high cell and collagen content and included also capillaries and arterioles, suggestive of a granulation tissue.

**Figure 5:**
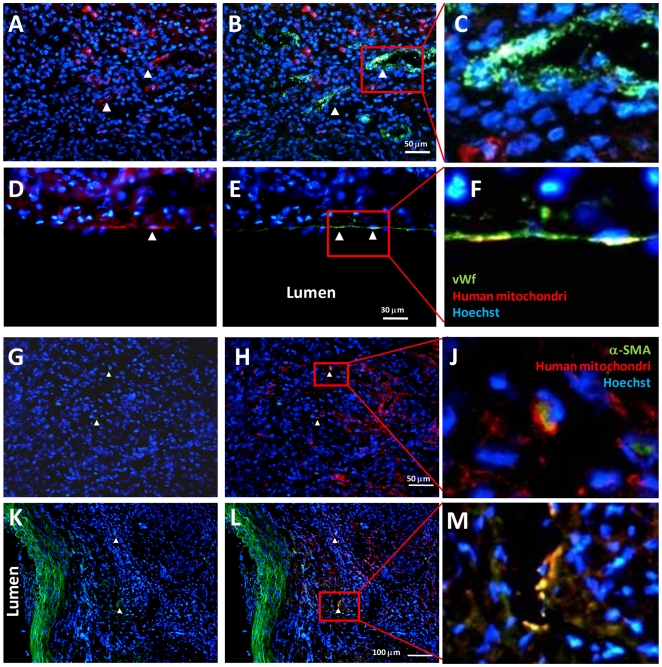
Origin of peritoneal patch remodeling cells. The remodeled patch developed as a new artery wall, with an endothelial layer. We asked whether cells that contributed to patch remodeling were of host (rat) or peritoneal (human) origin, by double staining with vWf (green in B–F) or alpha-SMA (green in H–M)) and human mitochondri (red). A–C) Within the densely cellulated reactive tissue that thickened the peritoneal patch, a few capillary-lining endothelial cells (green in B) were of human origin (red, higher magnification in panel C). D–F) Within the neo-formed endothelial layer (green in E), some cells (red in D) were of human origin (higher magnification in panel F). G–J) In the remodeled patch tissue, rare alpha-SMA staining cells (green in H and J) co-stained with human mitochondri (red). K–M) The same was in the close vicinity of the native aorta-peritoneal patch junction, where a medial smooth muscle layer (green in K, L) is still present.

**Figure 6:**
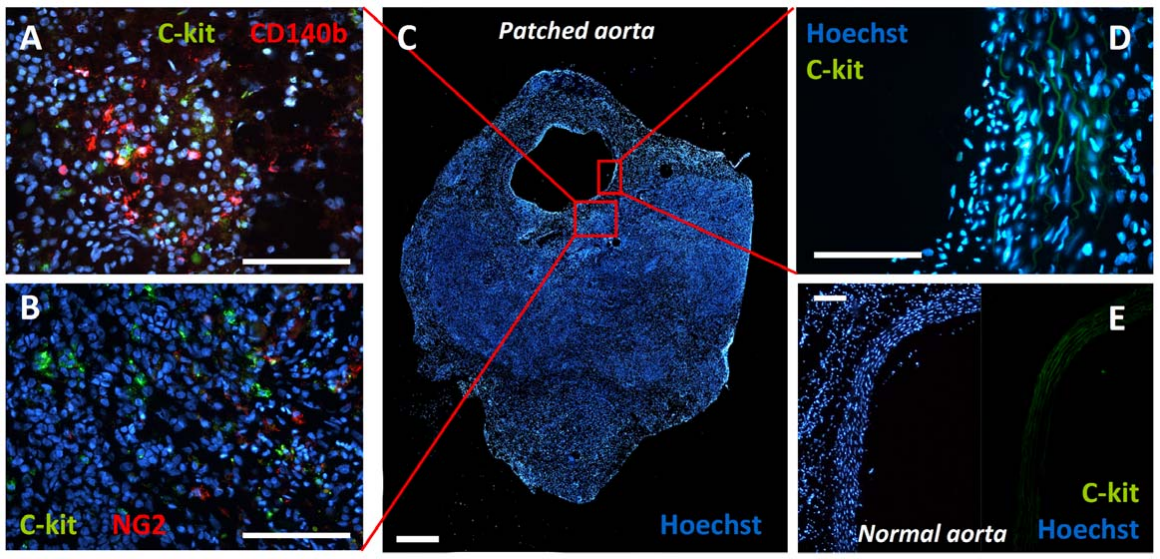
Progenitor cell markers in normal and patched aortic sections. A–C) Aortic sections including the peritoneal patch (C, scale bar 500 µm) were co-stained for c-kit and CD140b, also known as PDGFRB (A, scale bar 100 µm), or c-kit and NG2 (B, scale bar 100 µm). Panel D (scale bar 100 µm) shows c-kit staining of an endothelial area overlying the peritoneal patch (scale bar 100 µm). Panel E shows c-kit staining in a section of a normal aorta (scale bar 100 µm). In panel D and E, there is no c-kit signal from nucleated cells above the green background attributable to elastic fibers autofluorescence.

Most endothelial cells forming the intimal coverage of the patch were not of human origin, as demonstrated by colocalization immunofluorescence analyses showing rare overlap between vWf and human mitochondrial staining (Figure 5D–F). This finding suggests that extraperitoneal cells from the host local tissues or the bloodstream were responsible for the graft remodeling. Re-endothelization of a denuded arterial tract occurs through the contribution of local endothelial cells migrating from the edges of the lesion (in this case, the native aorta) and of circulating endothelial progenitor cells (EPCs) [18]. EPCs derive from the bone marrow and migrate into the bloodstream in response to vascular injury, where they selectively home to reconstitute anatomical and functional intimal integrity as well as to participate in angiogenesis [19]. Besides EPCs, circulating progenitors for the smooth muscle lineage have also been identified and may contribute to vascular remodeling and development of neointima [20]. In addition, several groups isolated progenitor cells for endothelium, smooth muscle and pericytes from the vessel wall itself, which represents a local reservoir for an optimal reaction to vascular injury [21], [22]. The present study was not designed to test whether remodeling occurred via local or circulating cells. Sparse c-kit+ cells were present in the tissue surrounding the patch, but there was no c-kit signal in the overlying endothelium and in the normal aortic wall (Figure 6), suggesting that the endothelium was not repopulated by circulating progenitors. In rats, c-kit may label both circulating and resident vascular progenitor cells [23], [24], while it has been shown that the vascular wall contains a population of pericytic progenitor cells which contribute to tissue remodeling [21], [25]. Some cells expressed the pericyte markers CD140b and NG2 in the patched aorta, but there was no co-expression of c-kit (Figure 6A–B), arguing against a progenitor cell origin of such pericytic cells in the granulation tissue.

The observation that most cells of the newly formed vessel wall were not of human origin suggests that the peritoneum served as a biological scaffold for vascular reconstitution.

Nonetheless, a few capillaries within the reactive tissue surrounding the patch were composed of human endothelial cells (Figure 5A–C). Colocalization of alpha-SMA and human mitochondri was extremely rare (Figure 5K–M). In principle, this indicates that peritoneal cells can contribute to vascular wall cells, but this contribution seems quantitatively negligible.

Although we are not aware of any systematic study of major histocompatibility complex (MHC) in peritoneal cells, it is generally held that this type of tissue has low or no MHC expression. When we repeated the human-to-rat xenograft experiments with rat immunosuppression by cyclosporine administration (daily dose of 5 mg/kg), histological appearance of the graft explants was identical, with the same degree of neo-intimal thickening and a similarly small contribution of human cells to the newly formed endothelium (not shown). This indicates that immune rejection was not the major process driving peritoneal patch remodeling and should not influence the cellular origin of cells in the remodeled aortic wall. Moreover, survival of animals with the peritoneal xenograft, absence of thrombosis and complete endothelization suggest that xeno-rejection did not occur in this model.

### Limitations and conclusion

This study has limitations inherent to the preliminary nature of the results, the relatively short observation period and the lack of a time-course study of the re-modeling process. However, for the first time we provide data indicating that the peritoneum can serve as a biological scaffold of a new vascular substitute. If further experiments in large mammals confirmed these findings, clinical trials in humans should be warranted.

## References

[1] Streinchenberger R, Barjoud H, Adeleine P, Larese A, Nemoz C (2000). Venous allografts preserved at 4 degrees C for infrainguinal bypass: long-term results from 170 procedures.. Ann Vasc Surg.

[2] Fahner PJ, Idu MM, van Gulik TM, Legemate DA (2006). Systematic review of preservation methods and clinical outcome of infrainguinal vascular allografts.. J Vasc Surg.

[3] Albers M, Romiti M, Pereira CA, Antonini M, Wulkan M (2004). Meta-analysis of allograft bypass grafting to infrapopliteal arteries.. Eur J Vasc Endovasc Surg.

[4] Hoenig MR, Campbell GR, Rolfe BE, Campbell JH (2005). Tissue-engineered blood vessels: alternative to autologous grafts?. Arterioscler Thromb Vasc Biol.

[5] Yung S, Li FK, Chan TM (2006). Peritoneal mesothelial cell culture and biology.. Perit Dial Int.

[6] Hernando A, Garcia-Honduvilla N, Bellon JM, Bujan J, Navlet J (1994). Coatings for vascular prostheses: mesothelial cells express specific markers for muscle cells and have biological activity similar to that of endothelial cells.. Eur J Vasc Surg.

[7] Mirza A, Hyvelin JM, Rochefort GY, Lermusiaux P, Antier D (2008). Undifferentiated mesenchymal stem cells seeded on a vascular prosthesis contribute to the restoration of a physiologic vascular wall.. J Vasc Surg.

[8] Sparks SR, Tripathy U, Broudy A, Bergan JJ, Kumins NH (2002). Small-caliber mesothelial cell-layered polytetraflouroethylene vascular grafts in New Zealand white rabbits.. Ann Vasc Surg.

[9] Campbell JH, Efendy JL, Campbell GR (1999). Novel vascular graft grown within recipient's own peritoneal cavity.. Circ Res.

[10] Pacholewicz JK, Daloisio C, Shawarby OA, Dharan SM, Gu J (1994). Efficacy of autologous peritoneum as a biological membrane in cardiac surgery.. Eur J Cardiothorac Surg.

[11] Sarac TP, Carnevale K, Smedira N, Tanquilut E, Augustinos P (2005). In vivo and mechanical properties of peritoneum/fascia as a novel arterial substitute.. J Vasc Surg.

[12] Sarac TP, Augustinos P, Lyden S, Ouriel K (2003). Use of fascia-peritoneum patch as a pledget for an infected aortic stump.. J Vasc Surg.

[13] Garcia-Graz NJ, Galindo-Ibarra JL, Garcia-Soto G, Mejia-Arreguin H, Trejo-Suarez J (2008). [Vascular graft of aponeurosis with peritoneum in dogs].. Cir Cir.

[14] Efendy JL, Campbell GR, Campbell JH (2000). The effect of environmental cues on the differentiation of myofibroblasts in peritoneal granulation tissue.. J Pathol.

[15] Gotloib L, Gotloib LC, Khrizman V (2007). The use of peritoneal mesothelium as a potential source of adult stem cells.. Int J Artif Organs.

[16] Rosellini A, Michelini M, Tanda G, Mandys V, Revoltella RP (2007). Expansion of human mesothelial progenitor cells in a longterm three-dimensional organotypic culture of Processus vaginalis peritonei.. Folia Biol (Praha).

[17] Huang W, Zhang D, Millard RW, Wang T, Zhao T (2010). Gene manipulated peritoneal cell patch repairs infarcted myocardium.. J Mol Cell Cardiol.

[18] Fadini GP, Agostini C, Sartore S, Avogaro A (2007). Endothelial progenitor cells in the natural history of atherosclerosis.. Atherosclerosis.

[19] Takahashi T, Kalka C, Masuda H, Chen D, Silver M (1999). Ischemia- and cytokine-induced mobilization of bone marrow-derived endothelial progenitor cells for neovascularization.. Nat Med.

[20] Albiero M, Menegazzo L, Fadini GP (2011). Circulating smooth muscle progenitors and atherosclerosis.. Trends Cardiovasc Med.

[21] Campagnolo P, Cesselli D, Al Haj Zen A, Beltrami AP, Krankel N (2010). Human adult vena saphena contains perivascular progenitor cells endowed with clonogenic and proangiogenic potential.. Circulation.

[22] Sainz J, Al Haj Zen A, Caligiuri G, Demerens C, Urbain D (2006). Isolation of “side population” progenitor cells from healthy arteries of adult mice.. Arterioscler Thromb Vasc Biol.

[23] Fadini GP, Sartore S, Schiavon M, Albiero M, Baesso I (2006). Diabetes impairs progenitor cell mobilisation after hindlimb ischaemia-reperfusion injury in rats.. Diabetologia.

[24] Caplice NM, Wang S, Tracz M, Croatt AJ, Grande JP (2007). Neoangiogenesis and the presence of progenitor cells in the venous limb of an arteriovenous fistula in the rat.. Am J Physiol Renal Physiol.

[25] Psaltis PJ, Harbuzariu A, Delacroix S, Holroyd EW, Simari RD (2011). Resident vascular progenitor cells–diverse origins, phenotype, and function.. J Cardiovasc Transl Res.

